# The Hospitals Inquiry

**Published:** 1900-07-28

**Authors:** 


### The Hospital

A JOURNAL OF

The Medical Sciences and Hospital Administration.

VOL. XXVIII.—No. 722.] EDITED BY SIR HENRY BURDETT, K.C.B., AND SOLOMON C. SMITH, M.D., M.R.C.P. [JULY 28, 1900.

### THE HOSPITALS INQUIRY.

Two letters which appeared in the *Times* last Monday in regard to the treatment of the sick in South Africa will no doubt tend to relieve some portion of the anxiety which has been aroused by Mr. Burdett-Coutts. Nevertheless, they do not in any way take away from the necessity for a full inquiry being made. The great thing is to get at the facts, and among the first points on which the country, full of anxiety about friends and relatives, will desire information will naturally be as to how far the things described by Mr. Burdett-Coutts are to be taken as a sample of the whole.

Sir Henry Burdett, taking the charges made, and analysing them *au pied de la lettre*, holds that for practical purposes the indictment was limited to the condition of affairs at Bloemfontein "in relation, not to one complete hospital even, but to the remnant of a field hospital which was divided up and transferred in part elsewhere, the remainder being left apparently as a receiving station merely," and he goes on to say, what is undoubtedly true, that the military receiving station at Bloemfontein criticised by Mr. Burdett-Coutts "never was, and was never meant to be, a fully-equipped hospital at all." We can easily imagine, however, that Mr. Burdett-Coutts might answer, "Precisely so, and the very essence of my charge is that, although it never was, or was meant to be, a fully-equipped hospital, it was used as one for weeks and weeks after Bloemfontein was occupied"; and nothing could show more markedly the necessity for some such commission of inquiry as has been appointed than the fact that two men, both of whom have the reputation of being thoroughly wideawake, should take such opposite views of the same thing. Sir Henry Burdett, however, raises the further point that the field hospital in question was a mere receiving hospital, that Mr. Burdett-Coutts has failed to show "that he ever endeavoured to ascertain whether the patients he saw at his first visit to the receiving station at Bloemfontein were the same, in whole or in part, as those he saw on the second and subsequent occasions," and that until he has definite evidence on this point "his charges must be held to be non-proven." Here, again, we clearly want the facts. Were these field hospitals being used as mere receiving hospitals or not? The point which Sir Henry Burdett especially insists on is that the charges brought against those responsible for the medical care of the sick and wounded "are mainly confined to one such receiving station at Bloemfontein," that the state of affairs at that hospital was of a temporary character, and was due to the unavoidable exigencies of war, and that the descriptions which have been given of affairs at Bloemfontein are not applicable to the general situation in South Africa, which is practically what Mr. Wyndham has already said.

Turning now to the long and interesting letter in which Professor Watson Cheyne reviews the medical history of the campaign from the arrival at Bloemfontein to the arrival at Pretoria, we find much to support Sir Henry Burdett's contention that the difficulty, as it actually existed, was a local one, due to the enormous rush of cases of enteric fever, to "exigencies of war," and to difficulties of transport. On the other hand it shows very fully that, although the trouble was local, it was very real and very serious; and second, that it was largely due to the fact that on the march from the Modder the equipment of the medical department was "cut down to a very great extent." "Owing to military exigencies these field hospitals had," he says, "arrived in Bloemfontein with very much reduced equipment, and they very soon became overcrowded with sick to the most distressing extent, and not only was there extremely imperfect accommodation for the patients, but both the medical staff and the orderlies were undermanned and very much overworked. As the epidemic continued to spread this sad state of affairs became daily more and more distressing, while there was but little increase in the facilities for dealing with it." Mr. Watson Cheyne does not hesitate to say that many of the difficulties and many of the defects which may be picked out in the Army medical service in the field depend on one great point, namely, that the Army Medical Department is not provided with its own independent transport. On the march from the Modder it appears that the ambulances and the bearer companies were reduced to one-fifth of their normal strength, and it goes without saying that "had these units arrived at Bloemfontein with five times the amount of material than that which they had" things would have been very different. Mr. Watson Cheyne goes on to say that the field hospital which has done the greatest amount of good work during the campaign is the New South Wales ambulance, which "came provided with its own transport, and formed a unit absolutely independent of the rest of the army," and adds that "in order that the Army Medical Department should be thoroughly efficient in the field they should have their own transport complete for all purposes, and irremovable. This is a matter which the Army Medical Department has been urging on the military authorities for some time." This, of course, is also a matter as to which there has always been much difference of opinion. We may, however, at least say that if the Medical Department does not have its own transport, those who "cut down" its equipment so largely are responsible for much that follows, and that one of the most important points which the Commission will have to investigate will be the relations existing between the medical and the transport departments, and that not merely in regard to field hospitals, but also as to the delay in bringing up the general hospitals, which were so badly wanted.

